# Current Understanding of the Pivotal Role of Mitochondrial Dynamics in Cardiovascular Diseases and Senescence

**DOI:** 10.3389/fcvm.2022.905072

**Published:** 2022-05-18

**Authors:** Yoshihiro Uchikado, Yoshiyuki Ikeda, Mitsuru Ohishi

**Affiliations:** Department of Cardiovascular Medicine and Hypertension, Graduate School of Medical and Dental Sciences Kagoshima University, Kagoshima, Japan

**Keywords:** mitochondrial dynamics, fission and fusion, ischemia-reperfusion, heart failure, hypertension, atherosclerosis, senescence, pulmonary hypertension

## Abstract

The heart is dependent on ATP production in mitochondria, which is closely associated with cardiovascular disease because of the oxidative stress produced by mitochondria. Mitochondria are highly dynamic organelles that constantly change their morphology to elongated (fusion) or small and spherical (fission). These mitochondrial dynamics are regulated by various small GTPases, Drp1, Fis1, Mitofusin, and Opa1. Mitochondrial fission and fusion are essential to maintain a balance between mitochondrial biogenesis and mitochondrial turnover. Recent studies have demonstrated that mitochondrial dynamics play a crucial role in the development of cardiovascular diseases and senescence. Disruptions in mitochondrial dynamics affect mitochondrial dysfunction and cardiomyocyte survival leading to cardiac ischemia/reperfusion injury, cardiomyopathy, and heart failure. Mitochondrial dynamics and reactive oxygen species production have been associated with endothelial dysfunction, which in turn causes the development of atherosclerosis, hypertension, and even pulmonary hypertension, including pulmonary arterial hypertension and chronic thromboembolic pulmonary hypertension. Here, we review the association between cardiovascular diseases and mitochondrial dynamics, which may represent a potential therapeutic target.

## Introduction

There were 55.4 million deaths worldwide in 2019, and 74% of those deaths were caused by non-communicable diseases, such as cardiovascular disease (CVD), stroke, cancer, diabetes mellitus, hypertension, and atherosclerosis. Accordingly, about half of the mortality rates due to non-communicable diseases are caused by CVD. There are 17.9 million deaths each year from CVD, which is estimated to be 32% of all deaths worldwide in 2019. These deaths include ischemia-reperfusion (I/R), heart failure (HF), hypertension (HTN), atherosclerosis, and pulmonary hypertension (PH) (1).

Since the heart consumes a lot of energy for rhythmic contraction, it highly depends on mitochondria, which produces a total of 95% of ATP in a cardiomyocyte by oxidative phosphorylation and thus plays a key role in cardiomyocyte responses to various conditions of stress induced by CVD (2). Mitochondria generate secondary reactive oxygen species (ROS) upon ATP production. Mitochondrial dysfunction due to various stresses increases secondary ROS, which is also responsible for the development of CVD, such as atherosclerosis, myocardial injury, and HF.

Mitochondria have dynamic states (fusion and fission) which can change their morphology to meet various cardiomyocyte functional demands. Mitochondrial fusion combines individual mitochondrial membranes by stimulation, and conversely, mitochondrial fission is marked by fragmentation of mitochondria and mitochondrial networks in response to stress, thus promoting removal of damaged mitochondria and the generation of new mitochondria (3). Mitochondria continuously bind by the fusion process and divide by the fission process. Accumulated evidence suggests that mitochondrial dynamics are associated with CVD and aging to maintain mitochondrial quality control. In this review, we summarize the current knowledge about the relationship between mitochondrial dynamics and CVD.

## Mitochondrial Dynamics in Cardiomyocytes

**Figure 1:**
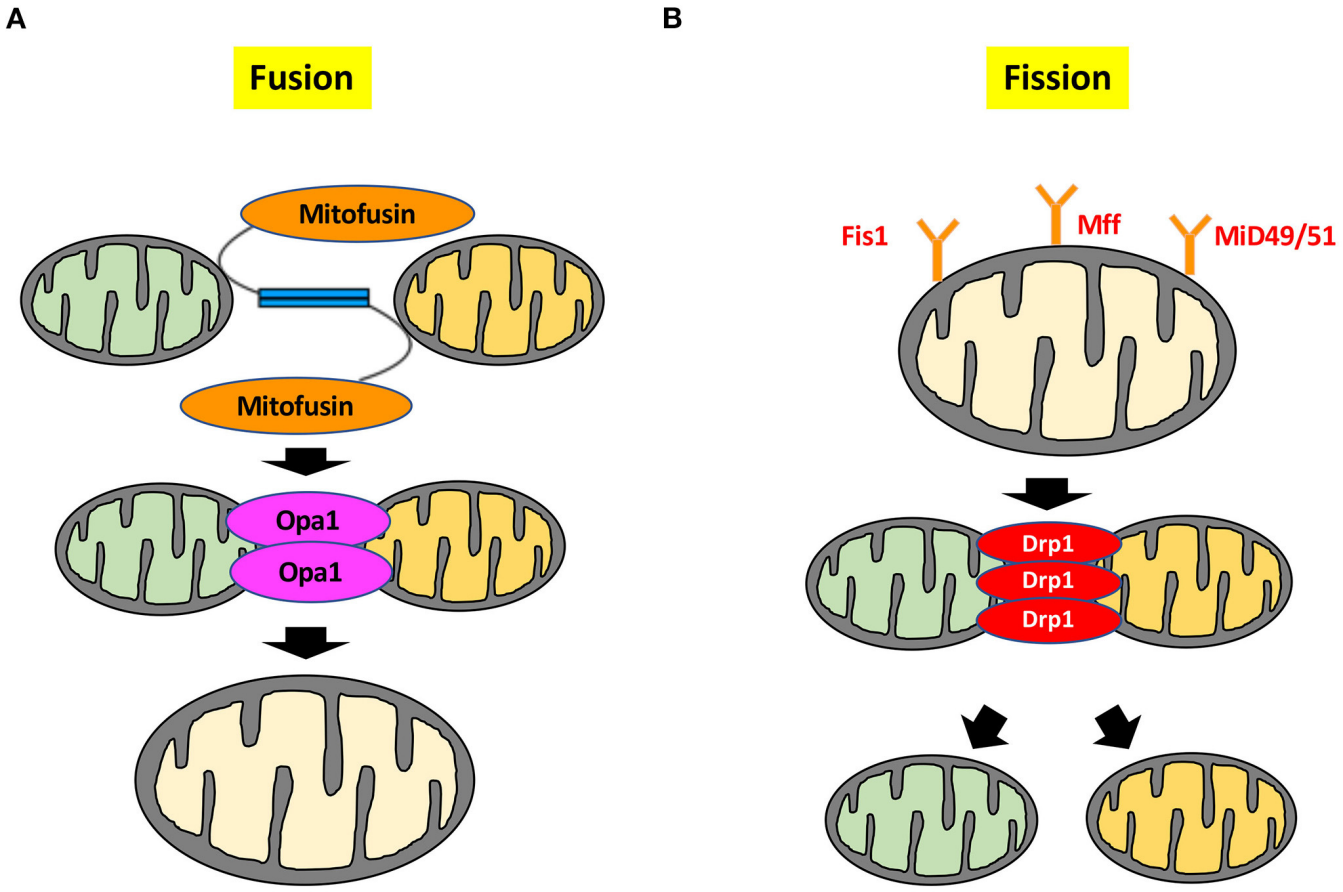
Molecular mechanisms of mitochondrial dynamics. **(A)** Mitochondrial fusion is controlled by Mfn1, Mfn2, and Opa1. Mfn1 and Mfn2 are located on the outer mitochondrial membrane, whereas Opa1 is located on the inner mitochondrial membrane to maintain the integrity of the cristae. **(B)** Mitochondrial fission is regulated by small GTPase Drp1. Drp1 is mainly localized in the cytosol, whereas Drp1 localizes to the outer mitochondrial membrane by several stimuli. Drp1 forms a complex with the outer mitochondrial membrane proteins Mff, Mid, and Fis1 to drive the mitochondrial fission process. Deformation of the mitochondrial membrane is caused by formation of the Drp1 complex, leading to mitochondrial division. Mid49/51; mitochondrial dynamics protein of 49 and 51 kDa.

Mitochondrial dynamics encompass the continuous processes of mitochondrial fusion and fission, which maintain a balance between mitochondrial biogenesis and turnover or apoptosis (Figure 1) (4). Mitochondrial fission generates small, spherical mitochondria, whereas fusion redistributes tubular or elongated mitochondria (3). They are controlled by mitochondrial fission and fusion proteins through opposing actions (5).

Mitochondrial fusion is controlled by small GTPases, such as mitofusin 1 (Mfn1), mitofusin 2 (Mfn2), and Optic atrophy-1 (Opa1). The 85kDa-GTPases Mfn1 and Mfn2 localize on the outer mitochondrial membrane, whereas the 100kDa-GTPase Opa1 is located on the inner mitochondrial membrane to maintain the integrity of the cristae (6, 7). Each of these fusion factors has a different role in cardiomyocytes. In cardiac-specific *Mfn1* knockout (KO) mice, mitochondria are smaller than those in wild type, but heart function and size are normal, and mitochondria still exert normal respiratory function (8). Mitochondrial fragmentation induced by *Mfn1* deletion is not sufficient to cause dysfunction of the cardiomyocytes and mitochondria. Conversely, the mitochondria of cardiomyocyte-restricted deletion of *Mfn2* are pleomorphic and enlarged. *Mfn2* KO mice have mild mitochondrial dysfunction and display modest cardiac hypertrophy and slight functional deterioration (9). These results indicate that Mfn1 and Mfn2 have different roles in regulating mitochondrial fusion. Mfn2 not only regulates mitochondrial fusion, but also plays a crucial role in mediating autophagosome-lysosome fusion in cardiomyocytes, which is necessary for mature autophagy, and is accompanied by autophagic degradation of damaged proteins and organelles, including mitochondria (10). Moreover, Mfn2 is phosphorylated by phosphatase and tensin homolog (PTEN)-induced kinase 1 (PINK1) and serves as a receptor for the cytosolic E3 ubiquitin ligase Parkin during mitochondria-targeted autophagy, which is called “mitophagy” (11). This mitophagy is essential for maintaining mitochondrial quality control by elimination of damaged mitochondria. Another role for Mfn2 is mitochondrial-endoplasmic reticulum tethering and Ca^2+^ transfer by engaging in homotypic and heterotypic complexes with Mfn1 or Mfn2 on the surface of mitochondria (12). Contraction of cardiomyocytes is also involved in Ca^2+^ handling through regulation of Mfn2. Cardiac contractions were not attenuated in *Mfn1/Mfn2* double KO mice treated with dobutamine. Whereas, Ca^2+^ transients were not influenced by *Mfn1/Mfn2* deletion, *Mfn2* deletion decreased mitochondrial Ca^2+^ uptake through reduction in cardiomyocyte sarcoplasmic reticulum-mitochondrial contact. Mfn2 regulated physical tethering of sarcoplasmic reticulum and mitochondria, which is essential for normal interorganelle Ca^2+^ signaling in cardiomyocyte (13). As described above, Mfn1 and Mfn2 are essential partners for outer mitochondrial membrane fusion by induction of mitofusin trans-interactions (14).

In addition to Mfn1/2, Opa1 is also mitochondrial fusion protein that maintains the structure of the cristae, which plays crucial role in aerobic cellular respiration and ATP synthesis (15, 16). *Opa1* KO induces ROS and mitochondrial dysfunction due to structural failure of the cristae, resulting in late-onset cardiomyopathy (17). *Opa1* homozygous KO is embryonic lethal in mice, while *Opa1* heterozygous KO mice die within 4 months (18). *Opa1* heterozygous KO mice induce fragmentation of mitochondria, disorganization of cristae structures, and impair mitochondrial respiratory function (17). In contrast, overexpression of *Opa1* promotes fragmented mitochondria, induces mitophagy, and increases antioxidant capacity, resulting in cardiomyocyte protection against oxidative stress through extracellular signal-regulated kinase (ERK) signaling (19). Taken together, normal mitochondrial fusion is essential to maintain normal cardiomyocyte function.

Mitochondrial fission is also regulated by small GTPases. The ~80 kDa GTPase dynamin-related protein-1 (Drp1) is a fission factor and is mainly located in the cytosol as a dimer or tetramer (20, 21). Drp1 is abundantly expressed in heart, skeletal muscle, kidney, and brain tissue (22). Drp1 is activated when various stimuli gather around the mitochondria from the cytoplasm and cause fission. When Drp1 is translocated from the cytosol to outer mitochondrial membrane, Drp1 interacts with the 17 kDa fission protein 1 (Fis1), mitochondrial fission factor (Mff), and mitochondrial dynamics proteins of 49 and 51 kDa to induce mitochondrial fission (23–26). Whereas, suppression of either Drp1 or Fis1 remarkably inhibits mitochondrial fission, overexpression of either *Drp1* or *Fis1* in cells promotes mitochondrial fission (27–29). However, Drp1 and Fis1 appear to have distinct roles. Drp1 mediated mitochondrial fission plays key role in the regulation of cristae remodeling (27). While overexpression of *Fis1* promotes the clustering of fragmented mitochondria around the nucleus, overexpression of *Drp1* results in fragmented mitochondria scattered throughout the cell (29). *Fis1* knockdown influences neither the recruitment of Drp1 to mitochondria nor mitochondrial fission in HeLa cells and HCT 116 cells. In cells with *Mff* knockdown, mitochondrial localization of Drp1 is reduced, and Drp1 is dispersed in the cytoplasm. Conversely, overexpression of *Mff* in cells induces mitochondrial fission, leading to an increase in Drp1 recruitment to mitochondria, indicating that Fis1 and Mff act as Drp1 receptors to facilitate mitochondrial fission (30). Fis1 also plays a crucial role in plasma membrane Ca^2+^-ATPase activity induced by the loss of subplasmalemmal mitochondria, indicating that mitochondrial fission is involved in regulation of cellular calcium homeostasis (29).

Interestingly, mice with cardiac-specific *Mfn1/Mfn2/Drp1* triple KO have a longer life and a unique pathological form of cardiac hypertrophy from that of *Mfn1/Mfn2* double KO mice. However, triple KO mice accumulate abnormal mitochondria over time, leading to distortion of sarcomere architecture in cardiomyocytes (31). On the other hand, sarcomere architecture of cardiomyocyte in *Mfn1/Mfn2* double KO mice is normal, indicating that Drp1 mediated mitophagy might be essential in maintaining normal sarcomere function (32).

From the above, mitochondrial dynamics play crucial roles in regulating cellular homeostasis. We summarized the role of mitochondrial dynamics in cardiomyocytes (Table 1). In the following section, we discussed the role of mitochondrial dynamics in response to CVD stress.

**Table 1:** Role of mitochondrial dynamics.

**Models**	**Species/tissue**	**Phenotype**	**References**
Cardiac-specific *Mfn1* KO	Mouse heart	Small mitochondria, Heart function and size are normal	(8)
*Mfn2* KO	Mouse cardiomyocyte	Mitochondrial dysfunction, cardiac hypertrophy	(9)
Mfn2	Mouse heart	Induction of autophagosome-lysosome fusion	(10)
Mfn2	HeLa cell	Mitochondrial-endoplasmic reticulum tethering and Ca^2+^ transfer	(12)
Mfn2	Mouse heart	Regulation of sarcoplasmic reticulum Ca^2+^ handling.	(13)
*Opa1* KO	Mouse heart	Mitochondrial dysfunction and structural failure of cristae	(17)
*Opa1* homozygous KO	Mouse	Embryonic lethal	(18)
*Opa1* heterozygous KO	Mouse	Death within 4 months	(18)
Overexpression of *Opa1*	Rat H9C2 cell	Cardiomyocyte protection	(19)
*Drp1* knockdown	HeLa cell	Attenuation of cristae remodeling and cytochrome c release during apoptosis	(27)
Overexpression of *Fis1*	HeLa cell	Promotion of clustering of fragmented mitochondria around the nucleus	(29)
Overexpression of *Drp1*	HeLa cell	Induction of fragmented mitochondria scattered throughout cell	(29)
*Mfn1/Mfn2/Drp1* KO	Mouse cardiomyocyte	Distortion of sarcomere architecture	(31)

## Mitochondrial Dynamics in Heart Diseases

### Mitochondrial Dynamics in Ischemia-Reperfusion Injury

Mitochondrial dynamics are closely related to I/R injury. Mitochondrial dynamics are changed remarkably following reperfusion, thereafter demonstrating a rapid and extensive fragmentation process in the mitochondria (9, 33). I/R injury allows Drp1 to translocate to outer mitochondrial membrane, causing excessive fission (34). I/R-induced mitochondrial fission is also derived from the decrease of Mfn1, Mfn2, or Opa1 expression levels, leading to decreased respiratory function which results in enhanced I/R-induced cardiomyocyte apoptosis (35–37). I/R-stress facilitates mitochondrial depolarization and induces secondary ROS, which attenuate mitofusins and Opa1 expression. I/R injury increases miR-140 which inhibits expression of *Mfn1* in cardiomyocytes, interrupts the mitochondrial network and exacerbates cardiomyocyte apoptosis (38). *In vitro* experiments using cardiomyocytes demonstrated that hypoxia upregulated Mfn2 (39). Other *in vivo* experiments showed that I/R injury increased Mfn2 (40). Cardiac-specific *Mfn2* KO mice under I/R show a great loss of mitochondrial membrane potential and significant decrease in survival ratio (10). In addition, cardiac-specific *Mfn1/Mfn2* double KO mice have a decreased infarct size in the myocardium in response to I/R (41). These results indicate that upregulation of *Mfn2* following I/R may be an adaptive cardioprotective response. Conversely, *Mfn2* KO delays mitochondrial permeability and transition pore opening, preventing I/R injury in cardiomyocytes (9). Hence, the role of Mfn2 against I/R injury is still controversial. Conversely, Opa1 plays protective role in cardiomyocytes following I/R. Opa1 expression was downregulated in cardiomyocytes with I/R injury *in vivo* and in hypoxia-treated cardiomyocytes *in vitro* (42). *Opa1* heterozygous KO mice induce more cardiomyocyte death under an I/R condition and subsequently exhibit greater infarct size in the myocardium after exposure to I/R than control (43). *Mfn1* overexpression significantly improves microvascular function under hypoxia conditions, while *Mfn2* overexpression protects cardiomyocytes from I/R injury (44, 45). *Opa1* overexpression induces mitophagy, which improves hypoxia-treated cardiomyocyte damage and cell viability (42). These results indicate that the enhancement of mitochondrial fusion factors has a protective effect on the ischemic heart.

On the other hand, detrimental effects of mitochondrial fission on cardiomyocytes with I/R is related to Drp1 activation. Administration of mitochondrial division inhibitor-1 (mdivi-1), a specific inhibitor of Drp1, prior to I/R inhibited mitochondrial fission and prevented the opening of the mitochondria permeability transition pore, decreasing cell death and infarct size in a murine model (46). Both mitochondrial calcium overload and oxidative stress under I/R conditions contribute to shifting mitochondrial dynamics toward fission (33). Increased Ca^2+^ is a potent regulator of Drp1 following reperfusion, and miR-499 can counteract the effects of Ca^2+^ overload on Drp1 activities (47, 48). Drp1 has five phosphorylation sites at serine 585, 616, 637, 656, and 693, of which, serine 616, serine 637, and serine 656 participate in regulating Drp1 activation/inactivation and mitochondrial fission in cardiomyocytes in I/R conditions (33, 49–53). Accumulation of Ca^2+^ in the perfused heart enhances the calcium-activated phosphatase calcineurin that dephosphorylates Drp1 at serine 637, leading to mitochondrial fragmentation and initiation of apoptosis (33). Pim-1 proto-oncogene, serine/threonine kinase suppresses the mitochondrial sequestration of Drp1 to sustain mitochondrial integrity and protect cardiomyocytes from I/R insult. Comparatively, protein kinase C δ contributes by facilitating phosphorylation activation of Drp1 at serine 616, and thus induces mitochondrial fragmentation (34, 54). Either inhibition of serine 637 phosphorylation by downregulation of Pim-1 or an increase of serine 656 phosphorylation by protein kinase A stimulates Drp1 activation to facilitate mitochondrial fission upon I/R (51, 55). Drp1 is also involved in diastolic function exacerbated by I/R. Dephosphorylation of Drp1 at serine 637 improved diastolic function in C57BL6/J I/R model (33). Inhibition of Thrombospondin 1 improved E/e' ratio exacerbated by I/R in aging rat model through inactivation of Drp1 (56).

From the above, the disturbance of mitochondrial dynamics is a key phenomenon in myocardial I/R injury which results in larger infarct volumes, cardiac cell death, and dysfunction. Either promotion of mitochondrial fusion and/or inhibition of mitochondrial fission may provide novel therapeutic targets to improve and reduce the impact of these injuries.

### Mitochondrial Dynamics in Cardiomyopathy

Mitochondrial fusion and fission are affected by metabolic signals (57). The balance between mitochondrial fission and fusion is regulated by changes in nutrient availability and metabolic demands, leading to adaptation of the mitochondria to changing conditions. C57BL/6 mice fed a high-fat diet experienced hyperlipidemia and hyperglycemia, and activated Drp1 by phosphorylation at serine 616, leading to induction of myocardial insulin resistance, contractile dysfunction and cardiomyocyte death (58). Lipid overload induced mitochondrial ROS and activated Drp1 by downregulating phosphorylation at serine 637 and upregulating phosphorylation at serine 616 by enhancing A kinase anchoring protein 121 degradation in lipotoxic cardiomyopathy (59).

Likewise, hyperglycemia in cardiomyocytes leads to Drp1-mediated mitochondrial fragmentation and thus increases ROS production. Alteration of mitochondrial energetics is closely associated with the development of diabetic cardiomyopathy. Compared to individuals without diabetes, patients with diabetes have reduced mitochondrial function in cardiomyocytes related to increased mitochondrial ROS and oxidative stress (60). Patients with diabetic cardiomyopathy have a decreased length of the intrafibrillar mitochondria in the heart. This morphological change is associated with a reduction in Mfn1 expression levels. Mfn1 expression is related to hemoglobin A1C, showing that hyperglycemia drives remodeling of the mitochondria (61). Hyperglycemia also induces the formation of short and small mitochondria in a rapid response by Drp1 (62, 63). Hyperglycemia activates phosphorylation of Drp1 at serine 616 and induces mitochondrial fission through Ca^2+^-mediated ERK1/2 signaling in cardiac myoblast cells (63). Cardiomyocytes in Zucker diabetic rats decrease the expression of Opa1 and Mfn2, and the phosphorylation of Drp1 at serine 637. Conversely, phosphorylation of Drp1 at serine 616 is increased, resulting in cardiomyocyte hypertrophy with abnormalities in mitochondrial dynamics and calcium handling through activation of the Orai1 calcium channel (64). Hyperglycemia suppresses the expression of Opa1 and Mfn1 and promotes that of Drp1 and Mfn2 in neonatal rat cardiomyocytes, decreasing mitochondrial membrane potential and increasing apoptosis (65). In human cardiomyocytes, an advanced glycation end product related to diabetes mellitus activates ERK1/2 and O-linked-N-acetyl-glucosamine glycosylation (66). This O-linked-N-acetyl-glucosamine glycosylation promotes expression of Opa1, decreases phosphorylation of Drp1 at serine 637, and contributes to mitochondrial fragmentation in a diabetic murine cardiomyocyte model (67, 68). Increased myocardial glucose decreases ATP production and myocardial glucose delivery by acute hyperglycemia developed mitochondrial dysfunction, causing contractile dysfunction in non-diabetic mice. Moreover, O-linked-N-acetyl-glucosamine glycosylation of the transcription factor specificity protein 1 causes glucose-dependent transcriptional repression, indicating that reduction of glucose utilization in diabetic cardiomyopathy might protect against glucotoxicity (69).

Drp1-mediated mitochondrial fragmentation is linked to insulin resistance in cardiomyocytes. Drp1-knockdown H9C2 cardiomyocytes exposed to H_2_O_2_ attenuate mitochondrial dysfunction and myocardial insulin resistance (70). Lipotoxic cardiomyopathy oversupplied by fatty acids is also related to insulin resistance, due to the accumulation of ceramide content, which increases expression levels of Drp1 and Mff (71).

Destruction of the mitochondrial quality control mechanisms regulating mitochondrial dynamics and mitophagy are related to dilated cardiomyopathy, which is characterized by systolic dysfunction and dilated ventricles (72). End-stage dilated cardiomyopathy is related to abnormally enhanced fragmentation of the mitochondria (73). Cardiac-specific *Mfn1/Mfn2* double KO mice induce development of cardiac dysfunction in 7 days, suggesting that inhibition of fusion and induction of unopposed fission of mitochondria may subsequently induce cardiac dysfunction (74, 75). Mitochondrial fission is stimulated during HF due to Ca^2+^ overload. Increased Ca^2+^ can be triggered by ROS and results in rapid and transient mitochondrial fragmentation (76). Cardiac expression of *Mfn1* is clinically reduced in non-responders who have cardiomyopathy that is resistant to current optimal, conventional, multidisciplinary therapies. In one study using neonatal rat ventricular myocytes, suppression of *Mfn1* decreased mitochondrial function by inhibiting mitochondrial respiration through the β-adrenergic receptor/cAMP/PKA/miR-140-5p pathway, resulting in metabolic remodeling and HF. This study indicates that *Mfn1* may be a clinical biomarker of non-responders in idiopathic dilated cardiomyopathy (77).

There have been various reports of drug-induced cardiomyopathy. Among them, doxorubicin-associated cardiomyopathy involves the regulation of mitochondrial dynamics. Treatment of FVB/N mice with doxorubicin for 4 weeks decreased expression levels of Mfn2 and increased those of Opa1 and Drp1. Doxorubicin also suppresses mitochondrial respiration and oxygen consumption, and accumulates autophagosomes due to impairment of the lysosomal degradation process (78). Septic conditions both *in vitro* and *in vivo* also contribute to mitochondrial dysfunction in cardiomyocytes. Sepsis decreases cardiac mitochondrial respiration and membrane potential while inducing ROS production and excessive mitochondrial fission through the interactions of Drp1 and Fis1 (79).

Chronic abnormal conditions in cardiomyocytes induce mitochondrial energetic dysfunction with disrupted internal structure, ROS-induced oxidative stress, and cell death. Suppression of mitochondrial fission decreases initial ROS production. While treatments for various cardiomyopathies are being gradually investigated, further research is needed to understand the mitochondrial dynamics of cardiomyopathy and to identify new treatment targets.

### Mitochondrial Dynamics in Heart Failure

Since the deletion of genes that regulate mitochondrial dynamics can cause HF *in vivo*, the normal functions of mitochondrial dynamics proteins may play key roles in heart protection from stress and eventual failure (80). For example, cardiac-specific *Drp1* heterozygous KO mice induced mitochondrial dysfunction and HF (81). Cardiac-specific *Yme1L* KO changed cardiac metabolism by reducing Opa1 levels through activation of Oma1, resulting in HF in mice (82). In another mouse model, cardiac-specific *Mfn2* KO increased the proportion of enlarged mitochondria and induced mitochondrial respiratory dysfunction (11). On the contrary, cardiac-specific overexpression of miR-122, which is elevated in HF patients, induced mitochondria-dependent cardiomyocyte apoptosis and accelerated HF through activation of Drp1 by inhibition of *Heart And Neural Crest Derivatives Expressed 2* (83).

**Figure 2:**
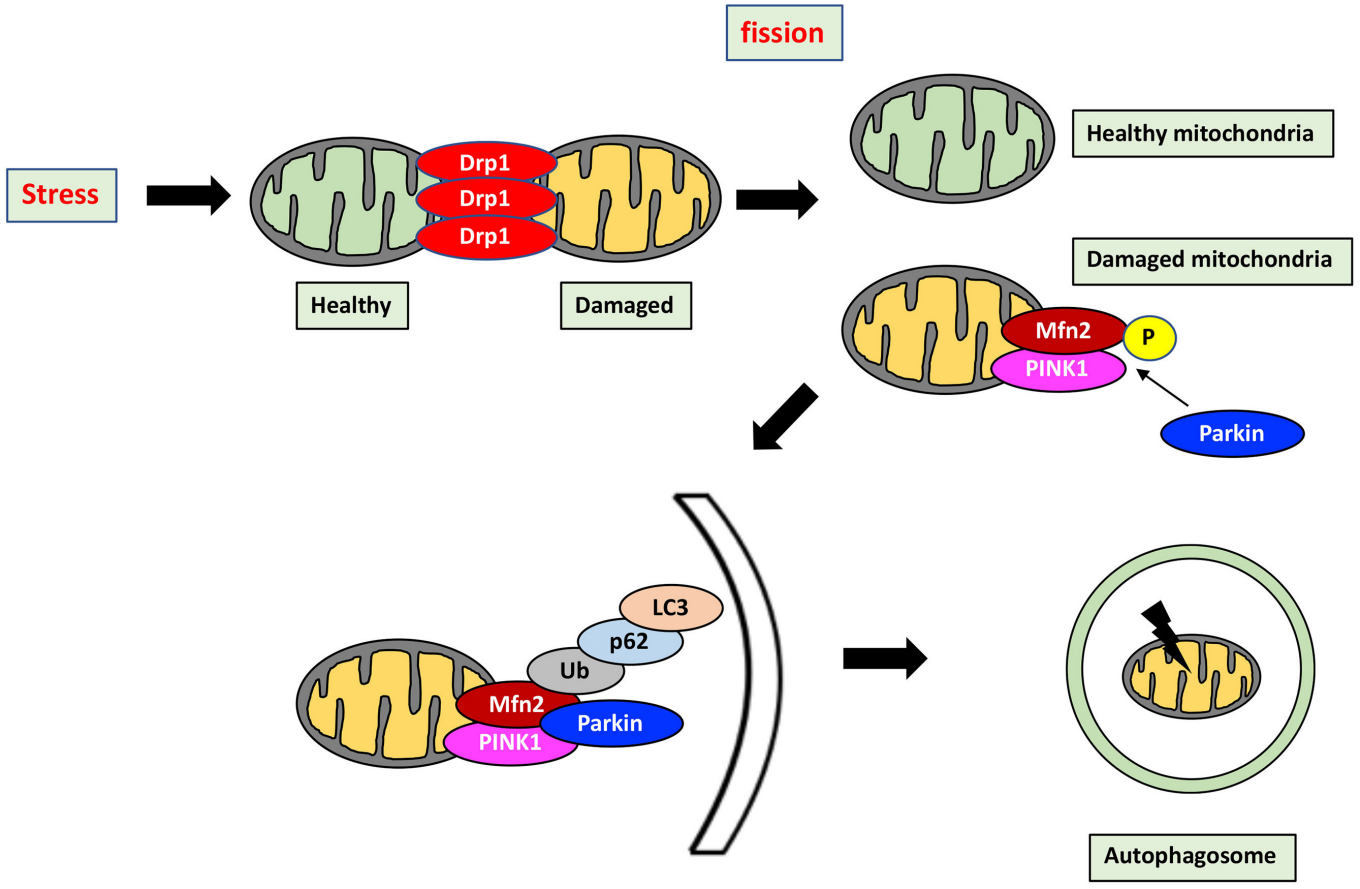
Mitochondrial quality control by mitochondrial dynamics and mitophagy. Mitochondrial health is maintained through the interaction between mitochondrial dynamics and mitophagy which is a selective separation of damaged mitochondria by autophagy. Stress induces mitochondrial fission, which divides stress-damaged mitochondria by healthy and damaged parts. Damaged mitochondria result in accumulation of PINK1, which phosphorylates Mfn2, in turn activating Parkin. Parkin then ubiquitinates Mfn2, and the Mfn2-Parkin interaction triggers mitophagy. P62 is recruited and binds the Parkin-ubiquitinated substrates, linking them to LC3. Mitochondria are then engulfed after elongation of the isolation membrane, referred to as the autophagosome. Mitophagy by the PINK1-Mfn2-Parkin signaling pathway is achieved by elimination of damaged mitochondria. P, phosphorylation; Ub, Ubiquitination.

Mitochondrial health in cardiomyocytes is maintained through not only mitochondrial dynamics but also mitophagy, which is the selective separation of damaged mitochondria by autophagy. Importantly, mitochondrial dynamics and mitophagy are closely correlated to each other (Figure 2). Indeed, Drp1 and Mfn2 play a key role in the induction of mitophagy. Mitophagy is transiently activated in mice with transverse aortic constriction, coinciding with mitochondrial translocation of Drp1. Ablation of *Drp1* caused downregulation of mitophagy which facilitated cardiac dysfunction (84). Several other studies also indicated that Drp1 mediated mitochondrial fission is essential for induction of mitophagy (85, 86). Mitophagy in cardiomyocytes is mediated by Parkin and PINK1. PINK1, in normal mitochondria, lacks stability and is quickly degraded. In contrast, PINK1 is stabilized in depolarized mitochondria, allowing its accumulation and facilitating Parkin recruitment from the cytosol to the depolarized mitochondria by Mfn2, a pivotal mediator of mitophagy through the PINK1-Mfn2-Parkin signaling pathway (87). PINK1 phosphorylates Mfn2, which activates Parkin (88, 89). Parkin then ubiquitinates Mfn2 (90). P62 is then recruited and binds the Parkin-ubiquitinated substrates, linking them to microtubule-associated protein light chain 3 (LC3) (91). Mitochondria are engulfed by elongated isolation membrane or Golgi body after elongation of the isolation membrane. This forms the autophagosome, resulting in degradation of the enclosed mitochondria (86, 91).

In this mechanism of mitophagy, PINK1 and Parkin play crucial roles in maintaining mitochondrial function so that insufficiency of mitophagy can induce damaged cellular homeostasis, causing cardiomyopathy and eventually HF. *PINK1* homozygous KO mice develop left ventricular (LV) dysfunction and pathological cardiac hypertrophy, which is mediated by increased oxidative stress and mitochondrial dysfunction in cardiomyocytes (92). Cardiomyocytes in a rat HF model exhibit increased ROS production derived from the mitochondria. In this model, Mfn2 and Drp1 expression are downregulated by about 50%, causing mitochondrial Parkin accumulation and induction of mitophagy. This attenuates mitochondrial damage through increased formation and consumption of ketone bodies, indicating that modulation of mitochondrial dynamics and mitophagy through Mfn2 and Drp1 regulation of ketone bodies plays crucial role in cardioprotection against HF (93). Clinically, however, the levels of PINK1 and Parkin are dramatically downregulated in HF (92, 93). Taken together, mitochondrial quality control through regulation of mitochondrial dynamics and mitophagy is extremely important in HF. HF is classified into HF with reduced ejection fraction (HFrEF) and HF with preserved ejection fraction (HFpEF). However, there are few reports relating mitochondrial dynamics to HFrEF and/or HFpEF. Relative to normal heart tissue, mitochondrial fission and cristae destruction are recognized in HFpEF, and these structural abnormalities in mitochondria are even more evident in HFrEF. Compared to normal heart tissue, HFrEF patient samples show increased Drp1 and decreased PGC-1α levels, while Mfn2 and Opa1 remain stable (94). Further investigation is needed to establish any relationship between mitochondrial dynamics, HFrEF, and HFpEF.

Ischemic HF is a chronic common disease but its relationship with mitochondrial dynamics is complex. As previously discussed, ischemic HF is associated with abnormalities in mitochondrial dynamics. Inhibition of mitochondrial fission reduces I/R injury in the heart (46). Opa1 expression is decreased both *in vivo* and *in vitro* under ischemic conditions (42). *Opa1* mutant heart tissue exhibits increased ROS and damaged mitochondrial function (18). These data suggest that suppressing mitochondrial fission or increasing the fusion induced by I/R may provide therapeutic targets.

In complex congenital heart disease patients, the right ventricle is subject to pressure overload through regulation of mitochondrial dynamics, resulting in right ventricular (RV) hypertrophy and eventually RV failure. In RV tissues of a RV failure mouse model, relative expression of Drp1 is increased, while that of Opa1 is decreased, indicating that regulating an optimal balance of mitochondrial dynamics may improve mitochondrial dysfunction and delay the development of RV failure (95).

Taken together, HF is characterized by increased mitochondrial damage, which may exacerbate cardiac dysfunction.

## Mitochondrial Dynamics in Vascular Diseases

### Mitochondrial Dynamics in Atherosclerosis

Atherosclerotic plaque is formed by lipid accumulation at sites of endothelial damage and dysfunction. The lipids are susceptible to oxidative modification by ROS, which are generated as a byproduct of the respiratory chain (96). Mitochondrial DNA (mtDNA) is vulnerable to damage because it lies close to the site of ROS production through activation of poly (ADP-ribose) polymerase 1, which is related to modulation of chromatin structure and DNA repair. Hence, mtDNA damage is an early event in atherogenesis (97, 98). Alteration in mitochondrial antioxidant enzyme levels, such as manganese superoxide dismutase, facilitates mtDNA damage in *apolipoprotein E* (*ApoE*) KO mice (97). Damaged mtDNA is observed in the circulating cells and hearts of patients with coronary artery disease, suggesting that mtDNA damage may contribute to the development of atherosclerosis (99). Recently, several studies reported the relationship between mtDNA and mitochondrial dynamics. mtDNA regulated stress-induced metabolic complementation through Opa1, and was lost by either *Drp1* inhibition or overexpression of *Opa1* (100–102). Major risk factors for atherosclerosis, including HTN, hyperlipidemia, smoking and diabetes mellitus, cause oxidative stress and induce inflammation (103, 104). An increase in risk factor-mediated oxidative stress lead to lipid peroxidation and mtDNA damage, resulting in mitochondrial dysfunction (104). Mitochondrial oxidative stress accelerated in high-fat-fed *Low-Density Lipoprotein receptor* homozygous KO mice and induced inflammation derived from macrophages through the nuclear factor-κB/monocyte chemotactic protein-1 pathway (105). However, whether dysregulation of mitochondrial dynamics could cause mtDNA damage remains unknown.

Vascular smooth muscle cells (VSMC) are closely related with plaque stability because they secrete the extracellular matrix which forms a fibrous cap. Migration and proliferation of VSMC cause the release of inflammatory factors that induce VSMC apoptosis, fibrous cap thinning, and subsequent plaque vulnerability (106). Inflammation and cell death are vital processes driving plaque development and transition to a vulnerable plaque phenotype (107). Thinning of the fibrous cap and induced necrotic core area are features of vulnerable plaques and were found in the bone marrow of *ApoE* KO mice. Isolated monocytes from *ApoE* KO mice revealed mtDNA damage and increased release of tumor necrosis factor alpha and interleukin-1 beta (96). These findings are linked to mechanisms of multiple diseases involving mitochondria and inflammation. Generally, VSMC proliferation and migration induced by inflammation result in pathological intimal hypertrophy of arteries (108). Angiotensin II (AngII), a major substrate of the renin–angiotensin system, induced VSMC proliferation and migration by regulation of mitochondrial fission and upregulation of mitogen-activated protein kinase kinase/ERK signaling and matrix metalloproteinase 2. Moreover, suppression of Drp1 by mdivi-1 attenuated VSMC proliferation and migration by blocking cytosolic and mitochondrial ROS production (109). Mfn2 also influenced VSMC proliferation and apoptosis. AngII increased VSMC proliferation through Mfn2 mediated Ras/Raf/mitogen-activated protein kinase kinase /ERK signal (110). Mfn2 levels were decreased in *ApoE* KO murine arteries, whereas overexpression of Mfn2 reduced VSMC proliferation (111). Platelet-derived growth factor-induced VSMC dedifferentiation decreased 50% of Mfn2 levels, resulting in induction of mitochondrial fission (112). Apelin-13, associated with induction of VSMC proliferation, increased expression of Drp1 and decreased that of Mfn1, Mfn2 and Opa1. Moreover, human aortic VSMC proliferation was attenuated by mdivi-1 (113). AngII also induced endothelial dysfunction, cellular proliferation, and inflammation, contributing to atherosclerosis through activation of protein kinase Cδ-dependent phosphorylation of Drp1 at serine 616 (109, 114). These results indicate that induction of mitochondrial fission and reduction of fusion may both be related to the pathophysiology of atherosclerosis.

Arterial media calcification is also one of the phenotypes of atherosclerosis, and Drp1 is involved in this pathological change. Osteogenic differentiation of human VSMC upregulated Drp1 expression. However, Drp1 inhibition by mdivi-1 during osteogenic differentiation attenuated matrix mineralization, cytoskeletal rearrangement, and mitochondrial dysfunction, and reduced type 1 collagen secretion and alkaline phosphatase activity, leading to attenuation of VSMC calcification both *in vitro* and *in vivo* (115). Moreover, lactate promoted Drp1 mediated mitochondrial fission through the nuclear receptor subfamily 4 Group A member 1/DNA-dependent protein kinase catalytic subunit /p53-pathway, and suppressed B-cell/CLL lymphoma 2 interacting protein 3-related mitophagy, resulting in arterial calcification (116).

Dynamic mitochondrial morphology is essential in the VSMC response to environmental stimuli. Alteration of ROS production resulting from damaged proteins, lipids, and DNA is associated with atherosclerosis. Either induction of mitochondrial fission and/or reduction of mitochondrial fusion by ROS production may cause mitochondrial dysfunction and apoptosis, leading to plaque progression in the arterial wall, VSMC proliferation, and arterial calcification.

### Mitochondrial Dynamics in Hypertension

As for the relationships among HTN, the vascular system, and mitochondrial dynamics, *Opa1* knockdown was reported to induce apoptosis in VSMC. Administration of L-NAME, a nonselective inhibitor of nitric oxide synthase, to *Opa1* heterozygous KO mice caused greater HTN compared to wild type (117). These *in vitro* and *in vivo* experiments indicate that Opa1 has a protective role against HTN by regulating endothelium-dependent relaxation to offset excessive ROS production. On the other hand, Drp1 activation is closely related to the development of HTN. Mitochondrial fission contributes to the VSMC phenotypic switch, which plays a crucial role in the pathogenesis of HTN. AngII-mediated mitochondrial dysfunction *in vivo* by inducing phosphorylation of Drp1 at serine 616, while Drp1 inhibition by mdivi-1 ameliorated mitochondrial dysfunction and prevented the AngII-induced VSMC phenotypic switch, leading to suppression of HTN (118). mdivi-1 reduced ROS production, cardiac hypertrophy and fibrosis in high-salt fed rats (119). Furthermore, mdivi-1 inhibited arterial constriction induced by endothelin-1, contributing to suppression of HTN (120). These results suggest that inhibition of mitochondrial fission by inactivating Drp1 may help to ameliorate HTN.

Other treatments against the adverse effects induced by HTN have been reported beyond direct manipulation of Drp1 or calcium sensing receptors (118–121). Poly(ADP-ribose) polymerase-inhibition prevented the development of LV hypertrophy in spontaneously hypertensive rats (SHR) through inactivation of Drp1 and activation of Opa1 and Mfn2 (122). Adventitial remodeling, the phenotypic switch of adventitial fibroblasts to myofibroblasts, is involved in HTN. Heat shock protein 90 inhibition by 17-dimethylaminoethylamino-17-demethoxygeldanamycin significantly suppressed AngII-induced mitochondrial fission and adventitial remodeling through the calcineurin/Drp1 pathway (123). Another report revealed that heat shock protein 90 inhibition also attenuated abdominal aortic aneurysm (AAA) in *ApoE* KO mice administered AngII (124). In fact, expression of Drp1 was increased in AAA tissues of both human sample and C57BL/6 administered with AngII and β-aminopropionitrile. Moreover, Drp1 inhibition attenuated the development of AAA size in *ApoE* KO mice treated with AngII. These results indicate that mitochondrial fission induced by Drp1 activation is involved in progression of AAA (125).

HTN contributes to the development of not only atherosclerotic cardiovascular diseases but also HF, through induction of adverse cardiac remodeling and LV hypertrophy (126). HTN-induced cardiac hypertrophy has been linked to changes in metabolic substrate utilization, dysfunction of the electron transport chain, and ATP synthesis (127). Decreased alpha-subunit of the mitochondrial precursor of ATP synthase was observed in LV hypertrophy in SHR (128). Mitochondrial fusion has a protective role against HTN-induced cardiac hypertrophy. The expression level of *Mfn2* was decreased in neonatal rat ventricular myocytes treated with phenylephrine, which contributed to the induction of cardiac hypertrophy (129). Calcium sensing receptor is also related to pathological cardiac hypertrophy through regulation of mitochondrial dynamics. Compared to wild type, SHR hearts exhibit significant upregulation of Drp1, whereas Opa1 and Mfn2 are downregulated. Calhex_231_, an inhibitor of calcium sensing receptor, ameliorates these mitochondrial dynamics changes, resulting in inhibition of apoptosis in hypertensive hearts (121). Interestingly, alpha1 receptor agonists affect mitochondrial fusion as well as fission, resulting in LV hypertrophy. Treatment of alpha1-adrenergic agonist phenylephrine decreased expression of Mfn2 in neonatal rat ventricular cardiomyocytes (129). On the other hand, other alpha1-adrenergic agonist norepinephrine induced mitochondrial fission through dephosphorylation of Drp1 at serine 637 in rat cardiomyocytes (130).

As a result of HTN, mitochondrial fusion is suppressed, whereas fission is induced. However, mitochondrial function in compensatory hypertrophied myocardium is controversial. Increased Mfn2 levels in SHR induced mitochondrial impairment. Pomegranate extract suppressed oxidative stress and alleviated mitochondrial function through activation of adenosine monophosphate-activated protein kinase (AMPK)/Nuclear factor-erythroid 2-related factor 2 signaling (131). Further research is needed on the role of Mfn2 in HTN. Interestingly, moderate exercise increased Opa1 and Mfn2 in wild type rats, whereas neither was affected in SHR, indicating that aerobic exercise may not be involved in modulating mitochondrial adaptive responses in patients with HTN; therefore, prevention of HTN is important to maintain mitochondrial function (132).

### Mitochondrial Dynamics in Pulmonary Hypertension

PH is defined by a mean pulmonary arterial pressure ≥25 mmHg and is characterized by severe disease with remodeling of the vasculature and increased resistance, stiffness, and fibrosis of the pulmonary artery. There are 5 types of PH depending on the location of lesions, and among these 5 groups, pulmonary arterial hypertension (PAH) and chronic thromboembolic pulmonary hypertension (CTEPH) are the major pathophysiological presentations of PH, as the pre-capillary type. RV failure is a major cause of death in patients with PH including PAH and CTEPH. Alterations in mitochondrial metabolism—notably impaired glucose oxidation—and increased mitochondrial fission, leading to RV dysfunction in PAH (133). Mitochondrial dynamics are involved in cell proliferation and apoptosis-resistant phenotypes in PAH. In PAH, the balance between fission and fusion is unstable due to increased activation of Drp1 and decreased expression of Mfn2; this tends to force mitochondrial dynamics toward fission (134). Overexpression of *Mfn2* increased mitochondrial fusion and reduced proliferation in pulmonary arterial SMC (PASMC), and inhibited proliferation of a medial thickness pulmonary artery in a monocrotaline-treated PAH model rat. Knockdown of phosphorylated *Mfn2* at serine 442 in PASMC from PAH patients attenuated proliferation, apoptosis, and cell cycle arrest through PINK1 and/or protein kinase A (135). Since Mfn2 is antiproliferative and proapoptotic, the increase in Mfn2 might contribute to improving hemodynamics against PAH (136). Unlike Mfn2, Mfn1 may contribute detrimentally to PAH. Mfn1 is increased both in hypoxia-induced PASMC and in mice in a hypoxic environment. miR-125a inhibited hypoxia-induced Mfn1 upregulation and PASMC proliferation, contributing to prevention of pulmonary vascular remodeling (137). Impaired RV function and fibrosis are related to upregulation of mitochondrial dynamics protein of 51 kDa and proglycolytic pyruvate kinase 2, contributing to promotion of ROS production and a glycolytic shift (138). Drp1 is also closely related to pulmonary vascular remodeling. Hypoxia inducible factor-1α activation in human PAH caused mitochondrial fission by cyclin B1/CDK1-dependent phosphorylation of Drp1 at serine 616. Inhibition of Drp1 by mdivi-1 improved PAH pathologically by arresting PASMC in the G2/M phase of the cell cycle (134). High-mobility group box-1 is identified as a biomarker of PAH pathogenesis due to its effect on pulmonary vascular remodeling by promotion of PASMC proliferation and migration. This occurs via activation of ERK/Drp1 mediated autophagy (139). Drp1 inhibition by mdivi-1 attenuated not only mitochondrial fragmentation but also endoplasmic reticulum stress in hypoxic PASMC (140). Under hypoxic conditions, apoptosis in PASMC was induced by ROS production through upregulation of Drp1 (141). One mechanism by which hypoxic conditions upregulate Drp1 is the induction of hypoxia inducible factor-1α. This pathway led to pulmonary vascular remodeling under hypoxia (142). Taken together these results indicate that Drp1 inhibition under hypoxic conditions may improve PAH. However, such treatment may not be a complete solution for PAH. In addition to PASMC, pulmonary arterial endothelial cells (PAEC) are involved in the pathophysiology of PAH. Whereas, hypoxia activated Drp1 and facilitated mitochondrial Ca^2+^-dependent proliferation and migration in PAEC, inhibition of Drp1 induced apoptosis resistance in PAEC, which may contribute to the development of PAH (143).

CTEPH is a severe cause of PAH. Abnormal VSMC phenotype switching is crucial to proliferative vascular remodeling in CTEPH. In the pulmonary vessels of patients with CTEPH, expression of phosphorylation of Drp1 at serine 616 was upregulated, whereas that of Opa1 was downregulated. Moreover, Wnt family member 5B contributed to VSMC phenotype switching in CTEPH through CAMKII-dependent phosphorylation of Drp1 at serine 616 (144). PAEC from patients with CTEPH had decreased expression of Mfn1, Mfn2 and Opa1, and increased ROS production (145). These results indicate that CTEPH tends to force mitochondrial fission in the pulmonary artery. Recent studies demonstrated that some miRNAs also regulate mitochondrial dynamics, and these miRNAs are involved in PAH, including CTEPH. We have recently been reported that the miR-140-3p level was higher and the miR-485-5p level was lower in patients with PAH and CTEPH than those patients in the control group without PH. Such alteration of miRNAs in our clinical study is thought to be related to the change of mitochondrial dynamics to fission. Additionally, miR-140-3p and miR-485-5p levels were related to hemodynamic parameters. Whereas, there was no relationship between miRNAs and SVO_2_ in either PAH or CTEPH, the miRNA-485-5P level was related to right atrial pressure. In addition, the miRNA-140 level was related to pulmonary vascular resistance and the miRNA-485-5P level was related to cardiac index in the PAH group. In the CTEPH group, the miRNA-485-5P level was related to right atrial pressure. These our findings suggest that mitochondrial dynamics-related circulating miRNAs may play pivotal roles in regulating hemodynamic changes in clinical PH (146).

From the above, mitochondrial fusion and fission proteins are potential targets to improve PAH. However, there are few reports on the relationship between mitochondrial dynamics and other types of PAH, such as PAH associated with connective tissue diseases, portal hypertension, HIV infection, and congenital heart diseases, necessitating further studies.

## Mitochondrial Dynamics in Cardiovascular Senescence

Human blood vessels undergo structural and functional changes with aging. Attention has recently been focused on mechanisms behind aging-mediated vascular dysfunction, and there are increasing reports on the relationship between aging and mitochondrial dynamics. Drp1 expression decreases with age, resulting in impairment of angiogenic function. Endothelial cell senescence promotes increased mitochondrial ROS through Drp1 regulation. Interestingly, inhibition of *Drp1* in young human umbilical vein endothelial cells induced mitochondrial dysfunction, ROS production, and angiogenic dysfunction, whereas overexpression of *Drp1* in senescent human umbilical vein endothelial cells improved autophagosome clearance and improved angiogenic function, leading to attenuation of angiogenic dysfunction (147). In VSMC, Suppression of Krüppel-like factor 5, transcription factor essential for cardiovascular remodeling, induced vascular senescence through induction of mitochondrial fission by increasing Drp1 and Fis1, while decreasing Mfn1 (148).

**Figure 3:**
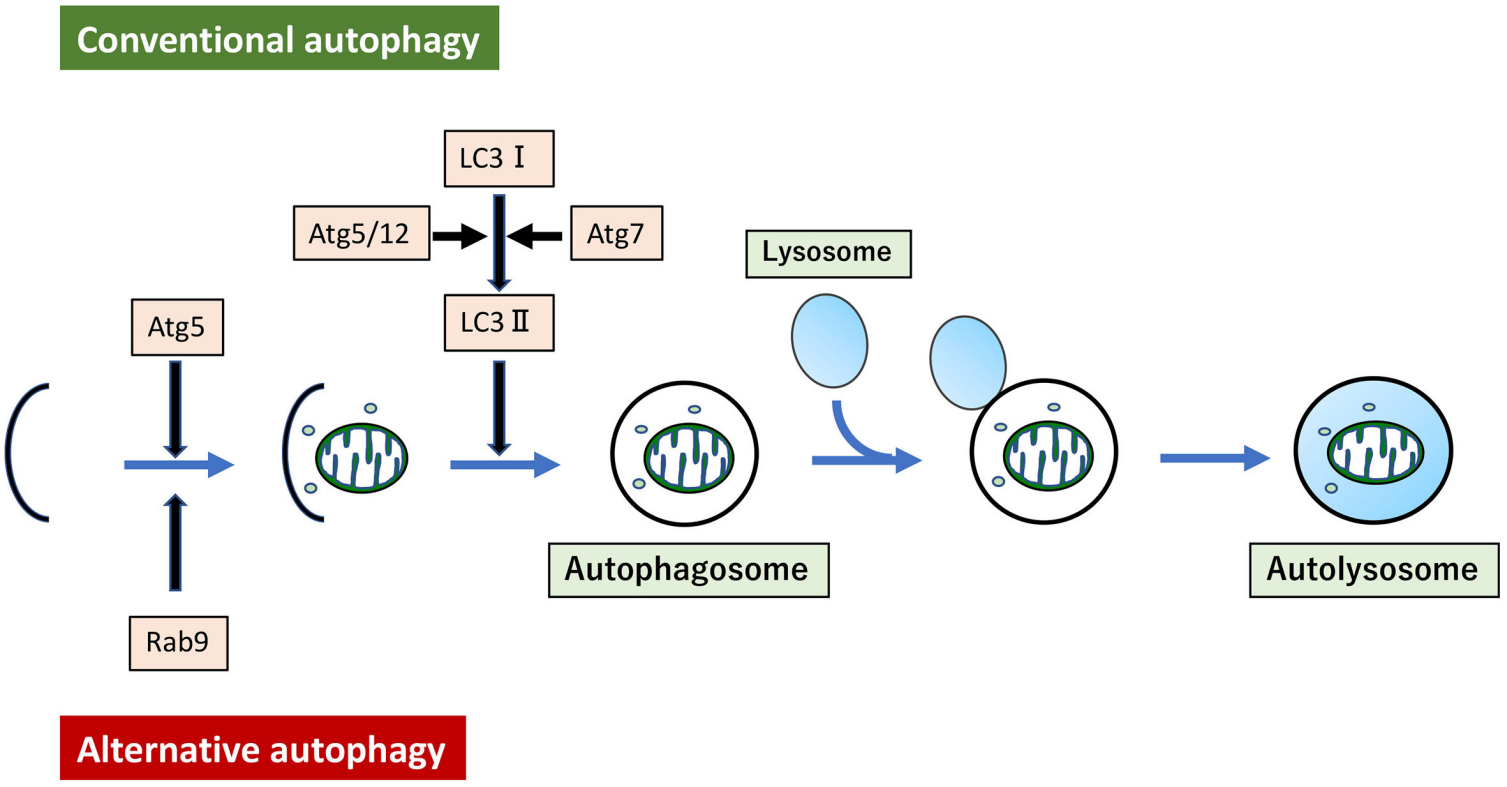
Conventional autophagy and Alternative autophagy. Mitophagy arises from two forms of autophagy. One is LC3-dependent conventional autophagy, which requires Atg5/Atg7, and the other is LC3-independent, membrane trafficking protein Rab9-dependent alternative autophagy.

Mitochondrial dysfunction can be induced in several ways. For instance, hyperlipidemia can induce mitochondrial dysfunction in arteries, leading to vascular senescence. Aging can also induce mitochondrial dysfunction through the elevation of interleukin-6 levels, associated with increased mitophagy derived from LC3 dependent autophagy (149). Treatment of VSMC with oxidized low-density lipoprotein (ox-LDL) induced mitochondrial fission through activation of phosphorylation of Drp1 at serine 616, and mitophagy derived from LC3-dependent autophagy (150). Although all autophagy described here so far has been LC3-dependent autophagy, recent studies show that mitophagy derived from LC3-independent and Rab9-dependent autophagy is also related to hyperlipidemia (151). Hence, the existence of at least two forms of autophagy have been reported. LC3-dependent conventional autophagy is well-described, while Rab9-dependent alternative autophagy is still novel (152) (Figure 3). In our experiments, administration of ox-LDL to VSMC or the arteries of *ApoE* KO mice conferred premature senescence accompanied by mitochondrial dysfunction. This included excessive mitochondrial fission induced by Drp1 activation via phosphorylation at serine 616. Either administration of mdivi-1 or AngII type I receptor (AT1R) inhibition ameliorated excessive mitochondrial fission, mitochondrial dysfunction, and premature cellular/arterial senescence through inactivation of Drp1 both *in vivo* and *in vitro*. The beneficial effect of AT1R on mitochondria and premature cellular/arterial senescence was caused by the inactivation of Drp1 thorough the inhibition of the AT1R/CRAF/mitogen-activated protein kinase kinase /ERK pathway. AT1R inhibition also upregulated mitophagy derived from Rab9-dependent autophagy, leading to the clearance of damaged mitochondria to maintain mitochondrial quality control. Eventually, AT1R inhibition also restored premature senescence in VSMC treated with ox-LDL and in the arteries of *ApoE* KO mice. (151). We have also reported the relationship between and mitophagy derived from Rab9-dependent autophagy and vascular senescence associated with estrogen deficiency. In estrogen-free VSMC and ovariectomized mice, treatment with 17β-estradiol induced mitophagy derived from Rab9-dependent autophagy through activation of the AMPK/ULK1/Rab9 pathway, leading to attenuation of mitochondrial dysfunction and vascular senescence induced by estrogen deletion. However, 17β-estradiol-induced mitophagy did not regulate mitochondrial dynamics factors (153). There are few reports on the relationship between alternative autophagy and mitochondrial dynamics, and thus the underlying mechanism is not clear.

Removal of senescent cells in aging mice attenuated atherosclerosis, indicating that vascular senescence is one of the strongest risk factors of pro-plaque formation (154). Therefore, it is very important to prevent progression of vascular senescence through improvement of vascular mitochondrial bioenergetics and inflammation. However, there are still few reports about the development of therapeutic strategies to improve senescence-associated mitochondrial dysfunction through regulation of mitochondrial dynamics, and thus further research is required.

## Treatments for Cardiovascular Diseases Through Regulation of Mitochondrial Dynamics

Recent several studies revealed that some kind of molecule and exercise may be relevant therapeutic tools. In a Wistar rat I/R model, aerobic exercise training increases levels of Mfn1 and Mfn2, resulting in decreased infarct size (155). Cordycepin also decreases infarct size in diabetic mouse models by inducing mitochondrial fusion through AMPK/Mfn2 pathway (156). Treatment of irisin induced Opa1-mediated mitophagy, which protected cardiomyocytes from further damage following myocardial infarction. *Opa1* knockdown attenuates the cardioprotective effects of irisin, leading to mitochondrial dysfunction due to increasing oxidative stress (42). Remote ischemic preconditioning increases the expression of Opa1 and decreases myocardial infarct size (35). Melatonin activates AMPK/Opa1 axis-mediated mitochondrial fusion and mitophagy, which improves cardiomyocyte death and mitochondrial dysfunction following I/R injury (157). Moreover, melatonin inactivates I/R injury-induced phosphorylation of Drp1 at serine 616 and induces mitophagy in type 2 diabetic rats through the Sirt 6/AMPK/peroxisome proliferators-activated receptor-γ co-activator-1α/Akt pathway (158). Hydralazine decreases the size of myocardial infarcts caused by cardiac I/R injury through inactivation of Drp1 (159). However, the inhibition of mitochondrial fission under I/R conditions should be approached with caution. Inhibition of mitochondrial fission by genetic deletion of *Drp1* enhances cardiomyocyte injury induced by I/R. In addition, mice treated with high dose mdivi-1, which suppressed Drp1 activation followed by fission, show a greater degree of myocardial damage by I/R than control mice. This is likely because of excessive inhibition of mitochondrial fission which suppresses mitophagy, thus resulting in accumulation of abnormal mitochondria (81). Therefore, further analysis is required to determine the optimal balance between mitochondrial fission and fusion. Interestingly, donepezil inactivates phosphorylation of Drp1 at serine 616, activates Mfn2 and Opa1, and induces mitophagy, resulting in improvement of ROS production, mitochondrial dysfunction, and cardiac apoptosis under I/R conditions. Donepezil may be an effective therapy to maintain the optimal balance of mitochondrial dynamics after I/R injury (160).

Regulation of the mitochondrial quality control mechanisms has also been associated with various types of cardiomyopathy. Recently, investigations have assessed possible treatments for such cardiomyopathies. Inhibition of Orai1-mediated Ca^2+^ entry into the mitochondria suppresses Drp1-mediated mitochondrial fission during hyperglycemia. This indicates that Orai1-inhibition may be a promising therapeutic target for diabetic cardiomyopathy by suppressing mitochondrial fission (64). Paeonol attenuates oxidative stress and apoptosis and improves cardiac hypertrophy and interstitial fibrosis. Paeonol activates Opa1-mediated mitochondrial fusion through the casein kinase II subunit alpha-signal transducer and activator of transcription 3pathway during diabetes *in vitro* and *in vivo* (161). There is one report that Klotho ameliorates doxorubicin-associated cardiomyopathy by suppressing phosphorylation of Drp1 at serine 616 (162). Apart from pharmacological treatment, exercise may also improve the function of diabetic cardiomyocytes. In a diabetic mouse model, exercise increases oxidative phosphorylation level and mitochondrial membrane potential but decreases ROS production and oxygen consumption, leading to improved blood pressure and reduced systolic dysfunction (163).

Maintaining the balance of mitochondrial dynamics and mitophagy may be a useful treatment target for various HF pathologies including I/R injury and cardiomyopathy. Sevoflurane postconditioning protects the heart from I/R injury by decreasing Opa1 levels and increasing Drp1 and Parkin, and stabilizes ATP levels by ameliorating mitochondrial impairment in rat hearts (164). Treatment with nicorandil suppresses fission and increases fusion through downregulation of Drp1 and upregulation of Opa1 and Mfn1 in a rat model of ischemic cardiomyopathy. Nicorandil also increases the opening of mitochondrial ATP-sensitive potassium channels, resulting in decreased myocardial pathological damage and apoptosis (165). Although many treatments for HF, such as selective inhibition of the Mfn1- beta II protein kinase C interaction, have been studied (166), questions relating to the mechanisms of function and pathogenesis remain, and further research is required.

Many studies suggest that certain foods, supplements, and drugs can regulate mitochondrial dynamics to treat atherosclerosis. A natural polyphenolic compound Resveratrol, found in grapes and red wine, increased Mfn1, Mfn2 and Opa1 expression, and attenuated damage caused to human umbilical vein endothelial cells by oxidative stress through the Tyrosyl-tRNA synthetase-Poly(ADP-Ribose) Polymerase 1 pathway (167). Dietary supplementation with fish oil prevented endothelial dysfunction in high-fat-fed *ApoE* KO mice through the induction of mitochondrial fusion by upregulation of Mfn2 and Opa1 (168). Ferulic acid, a bioactive component of rice bran, restored Mfn1 and Mfn2, which were decreased by high fat diet, and improved oxidative stress in high-fat-fed *ApoE* KO mice. The beneficial effects of ferulic acid were evident *in vitro* using human mononuclear cells under hyperlipidemia conditions (169). Coenzyme Q10 improved mitochondrial function, decreased ROS production, and promoted energy metabolism by activating the AMPK/YES-associated protein/Opa1 pathway, resulting in attenuation of atherosclerosis (170). AT1R inhibitor and adiponectin attenuated VSMC proliferation through Mfn2 mediated Ras/Raf/ERK signaling (110, 171). There are also some reports about arterial calcification. Melatonin and irisin attenuated arterial calcification by suppressing mitochondrial fission through AMPK/Drp1 signaling (172, 173). Quercetin, a type of polyphenol, also improved arterial calcification through inactivation of phosphorylation of Drp1 at serine 616 (174).

There are some reports detailing small molecules as treatments for PAH through the regulation of mitochondrial dynamics. Dichlorpacetate, a pyruvate dehydrogenase kinase inhibitor, improved RV fibrosis and hypertrophy in rats treated with monocrotaline. This occurred through the DNA methyltransferase 1/ Hypoxia inducible factor-1α/ pyruvate dehydrogenase kinase/ Drp1 pathway (175). Treatment with trimetazidine inhibited hypoxia-induced PASMC proliferation through downregulation of *Drp1* and upregulation of *Mfn2* (176). Liraglutide, a glucagon-like peptide-1 receptor agonist, inhibited the proliferation of PASMC via inactivation of the Drp1/nicotinamide adenine dinucleotide phosphate oxidase pathway and by LC3-dependent autophagy in PAH (177).

In this section, we summarized many treatments for CVD through the regulation of mitochondrial dynamics (Table 2). However, development of medical therapies targeting mitochondrial dynamics is still in the infancy. As described above, there are few reports for therapeutic target regarding of hypertension and vascular senescence so that we did not note in this section. It is imperative for future studies to investigate potential therapeutic targets and tools for treating and preventing CVD, and to determine the regulatory mechanisms of mitochondrial dynamics.

**Table 2:** Treatments of CVDs through regulation of mitochondrial dynamics.

	**Treatment/method**	**Species/cell**	**Effect of mitochondrial dynamics**	**Result**	**References**
**I/R**	RIPC	Wistar rat with I/R injury	Increase of Opa1	Decrease in MI size	(35)
	Irisin	Hypoxia-treated cardiomyocyte	Induction of Opa1-mediated mitophagy	Inhibition of apoptosis	(42)
	Aerobic exercise	Wistar rat with I/R injury	Increase of Mfn1 and Mfn2	Decrease in MI size	(155)
	Cordycepin	Diabetic mice with I/R injury	Increase of Mfn2	Decrease in MI size	(156)
	Melatonin	C57BL/6 with I/R injury	Increase of Opa1	Improvement of apoptosis and mitochondrial function	(157)
	Melatonin	Diabetic rat with I/R injury	Inhibition of p-Drp1 (Ser616)	Decrease in MI size and apoptosis	(158)
	Hydralazine	C57BL/6N with I/R injury	Drp1 inhibition	Decrease in MI size	(159)
	Donepezil	Wistar rat with I/R injury	Inactivation of p-Drp1 (Ser616) and activation of Mfn2 and Opa1	Improvement of apoptosis and mitochondrial dysfunction	(160)
**Diabetic CM**	BTP2	Zucker diabetic fat	Inactivation of p-Drp1 (Ser616) and activation of p-Drp1 (Ser637)	Improvement of cardiomyocyte hypertrophy	(64)
	Paeonol	Sprague-Dawley rat cardiomyocytes under high glucose condition	Increase of Opa1	Improvement of cardiomyocyte hypertrophy and interstitial fibrosis	(161)
**Dox-CM**	Klotho	C57BL/6 treated with doxorubicin	Inactivation of p-Drp1 (Ser616)	Suppression of apoptosis	(162)
**Ischemic HF**	Sevoflurane postconditioning	Sprague-Dawley rat with I/R injury	Increase of Opa1and Decrease of Drp1	Induction of mitophagy and improvement of myocardial ATP production	(164)
	Nicorandil	Rat with I/R injury	Decrease of Drp1and increase of Opa1 and Mfn1	Increase in opening of mitochondrial ATP-sensitive potassium channel	(165)
	SAMβA	Rat treated with AngII	Increase of Mfn1	Reduction of apoptotic cell death	(166)
**ATS**	ARB	Rat VSMC	Increase of Mfn2	Inhibition of cell proliferation	(110)
	Mdivi-1	Human VSMC	Drp1 inhibition	Attenuation of VSMC calcification	(115)
	Resveratrol	HUVEC treated with palmitic acid	Increase of Mfn1, Mfn2 and Opa1	Improvement of cell viability and reduction of oxidative stress	(167)
	Fish oil	High-fat-fed *ApoE* KO mice	Increase of Mfn1 and Opa1	Improvement of endothelial dysfunction	(168)
	Ferulic acid	High-fat-fed *ApoE* KO mice and Human mononuclear cell	Restored Mfn1 and Mfn2 which are decreased by high fat diet	Inhibition of oxidative stress	(169)
	Coenzyme Q10	High-fat-fed *ApoE* KO mice	Increase of Opa1	Inhibition of oxidative stress and promotion of energy metabolism	(170)
	Adiponectin	Human VSMC	Increase of Mfn2	Inhibition of cell proliferation	(171)
	Melatonin	Rat VSMC	Drp1 inhibition	Inhibition of arterial calcification	(172)
	Irisin	High-phosphorus-diet C57BL/6	Drp1 inhibition	Inhibition of arterial calcification	(173)
	Quercetin	Adenine-rich diet rat	Inhibition of p-Drp1 (Ser616)	Inhibition of arterial calcification	(174)
**HTN**	Mdivi-1	C57BL/6 treated with AngII	Drp1 inhibition	Inhibition of AngII-mediated phenotypic switch	(118)
	Mdivi-1	High-salt-fed rat	Drp1 inhibition	Inhibition of cardiac hypertrophy and fibrosis	(119)
	Mdivi-1	Rat VSMC	Drp1 inhibition	Inhibition of arterial constriction	(120)
	Calhex_231_	SHR	Restore of HTN-mediated Drp1 upregulation and Opa1/Mfn2 downregulation	Inhibition of apoptosis	(121)
	Pomegranate	SHR	Downregulation of Mfn2	Suppression of oxidative stress	(131)
**PH**	Dichlorpacetate	Monocrotaline-treated rat	Drp1 inhibition	Improvement of RV fibrosis and hypertrophy	(175)
	Trimetazidine	Human PASMC	Downregulation of Drp1 and upregulation of Mfn2	Inhibition of hypoxia-induced cell proliferation	(176)
	Liraglutide	Rat PASMC	Inactivation of Drp1	Inhibition of cell proliferation	(177)
**Senescence**	ARB	Human VSMC and *ApoE* KO mice	Inhibition of p-Drp1 (Ser616)	Attenuation of hyperlipidemia -induced senescence	(151)

*Ref, reference; ARB, Angiotensin II type I receptor inhibitor; MI, myocardial infarct; p-Drp1 (Ser616), phosphorylation of Drp1 at serine 616; p-Drp1 (Ser637), phosphorylation of Drp1 at serine 637; HUVEC, human umbilical vein endothelial cell; RIPC, remote ischemic preconditioning; CM, cardiomyopathy; Dox-CM, doxorubicin-associated cardiomyopathy; ATS, Atherosclerosis*.

## Perspective

Here we reported that mitochondrial dynamics plays a crucial role in CVD. Cardiac and vascular mitochondrial function reduces with age, contributing to the development of cardiovascular disease. As mentioned above, it could be considered that mitochondrial fusion is beneficial, and fission is detrimental, but this concept is not so clear. Although many CVDs are metabolic disease associated with mitochondrial dysfunction, mitochondrial dynamics might be an adaptive response rather than a causative factor in most of the CVDs. Mitochondrial fusion and fission contribute to the segregation and removal of impaired organelles. Depolarized mitochondria separated by mitochondrial fission are subsequently eliminated by mitophagy. Hence, mitochondrial fission is essential for inducing mitophagy to eliminate depolarized mitochondria. This is consistent with the report that strong inhibition of mitochondrial fission is rather harmful. Strong inhibition of Drp1 with high dose of mdivi-1 accumulated damaged mitochondria and facilitated cardiac damage by I/R (81). We also confirmed that high dose of mdivi-1 induced cellular senescence in VSMC treated with ox-LDL (151). In addition, mitochondrial fusion activator leflunomide used for the treatment rheumatoid arthritis has an adverse effect on humans, resulting in development of pulmonary hypertension (178).

**Figure 4:**
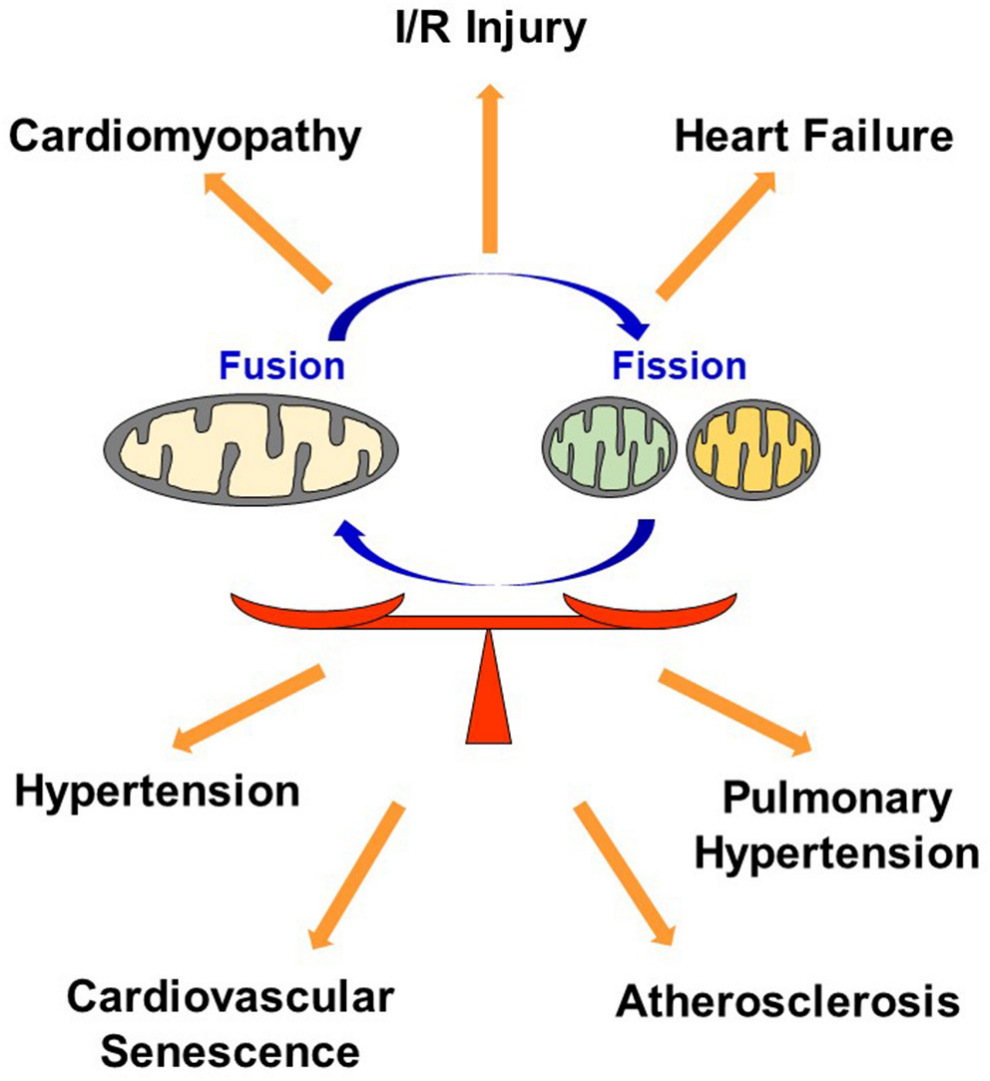
Dysregulation in mitochondrial dynamics causes CVD. Maintaining optimal balance of mitochondrial fusion and fission plays a key role in regulating the quality of cardiovascular homeostasis. I/R, Ischemia reperfusion.

In conclusion, dysregulation in mitochondrial dynamics has been implicated in many CVDs, and maintaining optimal balance of mitochondrial fusion and fission most likely plays a key role in regulating the quality of cardiovascular homeostasis and subsequent function (Figure 4).

## Author Contributions

All authors listed have made a substantial, direct, and intellectual contribution to the work and have approved it for publication.

## Funding

This work was supported by the Japan Society for the Promotion of Science KAKENHI (Grant Nos. JP19K07891 and JP21K15649).

## Conflict of Interest

The authors declare that the research was conducted in the absence of any commercial or financial relationships that could be construed as a potential conflict of interest.

## Publisher's Note

All claims expressed in this article are solely those of the authors and do not necessarily represent those of their affiliated organizations, or those of the publisher, the editors and the reviewers. Any product that may be evaluated in this article, or claim that may be made by its manufacturer, is not guaranteed or endorsed by the publisher.

## Abbreviations

CVD: cardiovascular disease
I/R: ischemia-reperfusion
HF: heart failure
HTN: hypertension
PH: pulmonary hypertension
ROS: reactive oxygen species
Mfn1: mitofusin 1
Mfn2: mitofusin 2
Opa1: Optic-atrophy 1
KO: knockout
ERK: extracellular signal-regulated kinase
PINK1: PTEN-induced kinase 1
Drp1: dynamin-related protein 1
Fis1: fission protein 1
Mff: mitochondrial fission factor
mdivi-1: mitochondrial division inhibitor-1
AMPK: Adenosine monophosphate-activated protein kinase-activated protein kinase
LV: left ventricular
HFrEF: heart failure with reduced ejection fraction
HFpEF: heart failure with preserved ejection fraction
RV: right ventricular
mtDNA: mitochondrial DNA
ApoE: apolipoprotein E
VSMC: vascular smooth muscle cell
AngII: angiotensin II
Angiotensin II type I receptor: AT1R
SHR: spontaneously hypertensive rat
PAH: pulmonary arterial hypertension
CTEPH: chronic thromboembolic pulmonary hypertension
PASMC: pulmonary arterial smooth muscle cell
PAEC: pulmonary arterial endothelial cell
LC3: Microtubule-associated protein light chain 3
AAA: abdominal aortic aneurysm
ox-LDL: oxidized low-density lipoprotein.
